# Enhanced recovery in the management of mild gallstone pancreatitis: a prospective cohort study

**DOI:** 10.1007/s00595-012-0364-9

**Published:** 2012-10-09

**Authors:** Xin Zhao, Da-Zhi Chen, Ren Lang, Zhong-Kui Jin, Hua Fan, Tian-Ming Wu, Xian-Liang Li, Qiang He

**Affiliations:** Department of Hepatobiliary and Pancreatospleenic Surgery, Beijing Chaoyang Hospital Affiliated to Capital Medical University, Beijing, 100020 China

**Keywords:** Enhanced recovery, Acute gallstone pancreatitis, Laparoscopic cholecystectomy, Endoscopic retrograde cholangiopancreatography

## Abstract

**Purpose:**

The aim of this study was to establish enhanced recovery protocols for the management of mild gallstone pancreatitis.

**Methods:**

Sixty consecutive patients were divided into enhanced recovery and traditional recovery (TR) groups in a randomized observational study. The basic enhanced recovery elements included early laparoscopic cholecystectomy, restrictive endoscopic intervention, and early oral nutrition. The incidence of complications, readmission, length of stay, and total medical cost were analyzed during the hospital course.

**Results:**

The length of hospital stay and medical cost were significantly lower in the enhanced recovery group in comparison to the TR group: 5.9 days vs. 10.6 days (*P* < 0.01) and ¥10,023 vs. ¥15,035 (*P* < 0.01). The complications and readmission rates in the two groups were similar.

**Conclusions:**

The implementation of enhanced recovery protocols is feasible in the management of mild gallstone pancreatitis. The utilization of these protocols can achieve shorter hospital stays and reduced costs, with no increase in either the re-admission or peri-operative complication rates.

## Introduction

Enhanced recovery (ER) pathways in surgical practices have recently reduced the duration of hospital stays and have been applied successfully in major surgery. They have been found to pose no risk to patients in terms of morbidity and mortality in colorectal [1], breast, and even pancreatic surgery [2, 3]. Although ER pathways could make an important contribution to distressed health care systems, they are used in less than one-third of surgical practices in the US and the UK [4, 5]. Acute pancreatitis may lead to a systemic inflammatory response syndrome with significant morbidity and mortality in 20 % of patients, thus ER practices are limited citing safety concerns.

“Gallstones” are the most common etiology of acute pancreatitis [6, 7]. Biliopancreatic obstruction is usually transient during the course of this disease because the offending stone passes rapidly into the duodenum; thus, acute pancreatitis is associated with minimal organ dysfunction and an uneventful recovery [8]. Therefore, the establishment of ER pathways could greatly reduce the time required for full recovery and could be applied to the management of mild acute gallstone pancreatitis (AGP). No previous randomized trial has focused on the adoption of ER principles such as the optimal time for cholecystectomy, use of enteral nutrition, and the choice of early endoscopic intervention in this subgroup of pancreatitis.

This study sought to establish protocols based on ER pathways for the treatment of mild AGP and to determine its safety, efficacy, and economic implications in a prospective cohort trail.

## Patients and methods

All patients who presented to the emergency ward within 24 h after the onset of acute pancreatitis from June 2009 to October 2011 were evaluated for study eligibility. The diagnosis of AGP was based on the presence of three criteria: (1) acute upper abdominal pain; (2) serum amylase more than three times the upper limit of normal combined with an increase in alanine aminotransferase (ALT) > 150 μ/l [9]; and (3) cholelithiasis and the characteristic findings of acute pancreatitis on admission CT scan or ultrasound. A diagnosis of AGP was established when 2 of 3 items were positive [10]. Patients with an APACHE II score <8 together with Balthazar CT score <4 were considered to have mild AGP. The exclusion criteria were an age below 18 years, pregnancy, patients with acute cholangitis, or with serious comorbid conditions that precluded laparoscopic cholecystectomy (LC).

Patients were randomized to receive either an ER pathway or traditional recovery (TR) care, using sealed envelopes. The envelopes were randomized in blocks and opened by a surgeon not otherwise engaged in the study. Randomization was performed once CT examination and laboratory studies had been completed, and the severity of pancreatitis was evaluated.

Supportive care in both groups included controlled fluid resuscitation, supplemental oxygen administered during the first 24–48 h and analgesia. Intravenous prophylactic antibiotics were administered to avoid the edema of duodenal papilla caused by acute inflammation during the early stage of mild AGP. Antibiotics were discontinued 5 days after hospitalization in the absence of the risk of pancreatic infection. No patient underwent gastrointestinal decompression or urinary catheterization before surgery. LC was performed during the initial hospital admission.

The TR group usually initiated early oral intake of limited calories when abdominal pain had subsided such that abdominal tenderness had markedly decreased, nausea and vomiting had ceased, and bowel sounds were present. The early use of endoscopic retrograde cholangiopancreatography (ERCP) was not restricted, in patients with jaundice, or known or suspected choledocholithiasis.

The contents of ER pathways mainly included early LC routinely on the second day after admission, restrictive ERCP before surgery, and early oral nutritional support. A special study surgeon provided the patients and their relatives with detailed information about their recovery program on the first day of hospitalization, to help build confidence that their postoperative pain would be reduced. Patients in the ER group underwent LC 2 days after admission. The restrictive indication of endoscopic intervention was the confirmation of common bile duct (CBD) stones combined with aggravated jaundice. The patients were allowed the early oral intake of carbohydrates from the day of admission. Table 1 compares the principles of management between the ER group and the TR group.

**Table 1:** Principles of enhanced recovery pathway and traditional care

	ER pathway	TR care
Preoperative support	Patients were given information on mild AGP and the ER pathway	Patients were informed in the standard manner
	Early oral intake of carbohydrates from the day of admission	Limited calories, usually initiated when abdominal pain had subsided
	Restrictive early endoscopic intervention	Early ERCP was not restricted, in patients with jaundice, or known or suspected CBD stones
	Provision of 10 % glucose (total 400 ml) 4 h before LC	No meals or water during the 8 h before surgery
Day of operation	Exploration of CBD, intra-operative cholangiography, or abdominal drainage was omitted	Exploration of CBD confirmed presence of stones before surgery, routine placement of abdominal drainage
	Liquid diet within 6 h after LC if no vomiting or nausea	Liquid diet 6 h after operation
	Patients encouraged to mobilize	No mobilization
Postoperative care		
Day 1 after surgery	Oral intake of normal diet	Low fat diet
	Expand mobilization	Start mobilization
Day 2 after surgery	Evaluate discharged criteria	Oral intake of normal diet, expand mobilization, and assess discharge criteria

*ER* enhanced recovery, *TR* traditional recovery, *AGP* acute gallstone pancreatitis, *ERCP* endoscopic retrograde cholangiopancreatography, *CBD* common bile duct, *LC* laparoscopic cholecystectomy

The discharge criteria were the same for the two groups: tolerance to solid diet; relief of abdominal pain; no surgical complications; and normal direct bilirubin level.

The primary endpoint was the intention to treat. Dichotomous variables were recorded as the absolute frequencies (number of cases) and relative frequencies (percentages). Continuous variables were recorded as the means and standard deviations (SD) or the median plus maximal and minimal values. Continuous variables were compared using two-sample *t* test when the data were normally distributed. The Wilcoxon rank sum test was used in the absence of normal distribution. Categorical data were analyzed using the Chi-square or Fisher exact test as appropriate. *P* < 0.05 was considered to be significant. All analyses were conducted using SPSS version 13 software package.

## Results

Of the 202 available gallstone pancreatitis patients, 60 were eligible for inclusion. No deaths occurred during the observation period in either group. The ER and TR groups were comparable in terms of age, gender, and severity of AGP (Table 2).

**Table 2:** Overview of the patient characteristics and surgery

	ER (*n* = 30)	TR (*n* = 30)	*P* value
Age (years)	56 (35–81)	58 (28–79)	0.30
Gender ratio: male/female	13/17	12/18	0.79
APACHE II score	3.2	3.3	0.44
CT severity index	0.9	1.1	0.14
ALT (μl)	225.1	198.1	0.13
DBil (μmol/l)	35.8	37.4	0.38
CRP (mg/l)	35.4	36.8	0.37
Type of surgery: LC/LC converting to open	29/1	27/3	0.61

*ER* enhanced recovery, *TR* traditional recovery, *ALT* alanine aminotransferase, *DBil* direct bilirubin, *CRP* C-reactive protein, *LC* laparoscopic cholecystectomy

There were no significant differences between the two groups in the levels of ALT, direct bilirubin, and C-reactive protein (CRP) on admission. All patients underwent cholecystectomy during the initial hospitalization. There was no significant differences between the ER and TR groups in the incidence of conversion of LC to an open procedure (*P* = 0.61). The exploration of the CBD was performed in one (3.3 %) of 30 patients in the ER group and five (16.7 %) of 30 patients in the TR group. Only one patient in the ER group underwent preoperative ERCP under the restrictive criteria. Endoscopic intervention was used in 11 patients in the TR group and detected that CBD stones in seven cases.

Table 3 shows the medical complications, readmission rate, hospital stay, and total hospital cost. The rate of readmission was 6.67 % in the ER group, in comparison to 3.33 % in the TR group (*P* = 1.00). Some patients in both groups were readmitted for residual stones in the CBD. The most common complication was acute lung injury. The complication rate was similar in the two groups and no surgical complications occurred. There was a significant difference between the two groups in the mean duration of hospital stay (*P* < 0.01), mainly due to the number of preoperative days (2.4 vs. 6.8 days, *P* < 0.01). Most patients in the two groups recovered rapidly after surgery and were discharged within 4 days (*P* = 0.30). The total hospital cost in the ER group was significantly less than that in the TR group (*P* < 0.01) due to the shorter hospital stay, fewer medical treatments were required during the preoperative period and less use of ERCP.

**Table 3:** Complications, cost and duration of hospital stay

	ER (*n* = 30)	TR (*n* = 30)	*P* value
Complications	1	2	1.00
Readmission	2	1	1.00
Duration of hospital stay (day)	5.9	10.6	<0.01
Days before operation (day)	2.4	6.8	<0.01
Days after operation (day)	3.7	3.5	0.30
Total hospital cost (¥)	10023	15035	<0.01

*ER* enhanced recovery, *TR* traditional recovery

## Discussion

Enhanced recovery pathways involve various techniques for the care of patients undergoing elective surgery. The methods used include minimally invasive procedures, optimal pain control, and aggressive postoperative rehabilitation, including early oral nutrition and ambulation. The present study indicated that a policy of ER in patients with mild AGP can result in a significantly reduced length of hospital stay and total cost with no increase in complications, readmission rate, or mortality.

The shorter hospital stay in the current series was mainly due to early LC, restrictive endoscopic intervention, and oral nutritional support initiated as early as possible.

Recurrent episodes of acute pancreatitis occur in as many as 30–50 % of patients with gallstone pancreatitis during the waiting period before interval LC. The guidelines recommend that all patients with biliary pancreatitis should undergo definitive management of gallstones during the same hospital admission, unless a clear plan has been made for definitive treatment within the next 2 weeks [5]. However, the timing of cholecystectomy in AGP remains controversial. The mean length of the hospital stay was reduced by 5 days in the ER group in comparison to the TR group, due largely to a reduction in the preoperative waiting time. David et al. concluded that a policy of early cholecystectomy in patients with mild-to-moderate gallstone pancreatitis resulted in a significantly reduced length of hospital stay with no increase in complications or mortality [11]. Rajeev supports the concept that early LC should be preferred in all patients with mild acute biliary pancreatitis (ABP) because it protects against further attacks of ABP during the wait for interval cholecystectomy [12]. In addition, the rate of conversion from laparoscopic to open cholecystectomy is greater in interval LC than in early LC. The current study found that the rate of conversion to an open procedure was less in the ER group than in the TR group, although the difference was not statistically significant. Routine intraoperative cholangiography was omitted in the ER group, because the incidence of bilo-pancreatic complications in patients with mild gallstone pancreatitis who undergo cholecystectomy is very low [13].

Endoscopic retrograde cholangiopancreatography is a challenging endoscopic procedure, with a reported rate of procedure-related complications of approximately 5–10 % [14, 15]. The need for early endoscopic intervention is controversial regarding patients with AGP [16]. Charles et al. believes that all cases of suspected gallstones pancreatitis with known or suspected choledocholithiasis should undergo systematic biliary sphincterotomy [17]. In contrast, Alejandro et al. reported that early endoscopic intervention failed to reduce systemic and local inflammation in patients with AGP and biliopancreatic obstruction [18]. Early endoscopic intervention is not required if acute cholangitis can be safely excluded and should not be considered a standard indication. Recent studies have demonstrated a 71–88 % rate of spontaneous disobstruction within 48 h after the onset of ABP (and a subsequent uneventful course of acute pancreatitis) [19, 20]. Indeed, only a small subgroup of patients might have a theoretical justification for ERCP. Several clinical studies have shown that the duration of obstruction (>48 h) is a critical determinant for ERCP [21]. The current series demonstrated that the levels of direct bilirubin decreased to normal spontaneously without emergent endoscopic intervention in 98 % (29/30) of patients in the ER group. Early ERCP should be limited in mild AGP. The indication for endoscopic intervention should be confirmed CBD stones combined with aggravated jaundice. The length of hospital stay can be shortened by avoiding the risks of early endoscopic intervention.

“Pancreatic rest” or reduction of exocrine secretion is very important in the treatment of patients with acute pancreatitis. Enteral nutrition has no positive impact on the course of mild acute pancreatitis and it is only recommended in patients who cannot consume normal food after 5–7 days [22]; as a consequence, several days of fasting is widely employed in most of these patients. However, the concept of pancreatic rest remains theoretical and has been inadequately tested; there are no randomized studies demonstrating that this practice hastens recovery in acute pancreatitis. Of the three main groups of nutrients (proteins, lipids, and carbohydrates), carbohydrates have the weakest stimulatory effect on pancreatic secretion [23]. The current study initiated the oral intake of carbohydrates on the day of admission, once abdominal pain had subsided, to shorten the fasting time in the ER group. No relapses of pain occurred within 48 h of the start of oral feeding. This change in approach in comparison to TR care may be implemented in AGP to shorten the preoperative period.

## Conclusions

The present study indicates that ER pathways can be implemented in the management of mild gallstone pancreatitis. Such protocols can lead to shorter hospital stays and reduced cost, with no increase in either the re-admission or peri-operative complication rates.

## Abbreviations

ABP: Acute biliary pancreatitis
AGP: Acute gallstone pancreatitis
ALT: Alanine aminotransferase
CBD: Common bile duct
CRP: C-reactive protein
ER: Enhanced recovery
ERCP: Endoscopic retrograde cholangiopancreatography
LC: Laparoscopic cholecystectomy
TR: Traditional recovery
